# Diagnosis and rehabilitation attempt of a patient with acquired dyslexia

**DOI:** 10.1590/S1980-57642008DN10100014

**Published:** 2007

**Authors:** Maria Teresa Carthery-Goulart, Mirna Lie Hosogi Senaha

**Affiliations:** 1 PhD, Cognitive and Behavioral Neurology Group, University of São Paulo School of Medicine, São Paulo, Brazil.

**Keywords:** acquired dyslexia, diagnosis, language and speech disorder rehabilitation, aphasia, dislexia adquirida, diagnóstico, reabilitação dos transtornos da fala e da linguagem, afasia

## Abstract

Although dyslexia is a common consequence of brain damage there are few studies about therehabilitation of this disorder. Cognitive Neuropsychology models of reading have been used to describe severalsyndromes of acquired dyslexia. Phonological dyslexia is a reading disorder characterized by a dysfunctionalgrapheme-to-phoneme conversion procedure, which affects the ability to read low frequency words and nonwords. Lexical reading is preserved and patients can read frequent words (regular and irregular).

**Objective:**

Verify the application of the cognitive model in the characterization of the reading disorder of a patient with acquired dyslexia and in the devising and implementation of a rehabilitation plan.

**Methods:**

This study presents OCS, a 57-year-old patient who suffered from acquired phonological dyslexia after a left temporo-parietooccipital ischemic stroke. A rehabilitation program based on the principles of Cognitive Neuropsychology was devised non-words and low frequency words with controlled lengths were used and the patient was stimulated to read them aloud in a 22-session treatment.

**Results:**

The post-test evaluation showed quantitative and qualitative improvements Significant improvements were verified in the total number of correct responses including self-correction attempts (p<0.01) and in the reading of trisyllabic and polysyllabic non-words of simple syllabic structure (p=0.0007 and p=0.02 respectively).

**Conclusions:**

The use of the cognitive model to devise a rehabilitation program was successful and we observed significant improvement of reading skills in a short period of treatment. The achievements over this period provided the patient with functional reading performance.

## Abstract (pt)

Embora a dislexia seja uma seqüela comum após lesões cerebrais há poucos estudos sobre a reabilitação deste distúrbio. Modelos de leitura da Neuropsicologia Cognitiva têm sido utilizados para descrever diversas síndromes de dislexia adquirida. A dislexia fonológica é um distúrbio de leitura caracterizado por disfunção do procedimento de conversão grafema-fonema que afeta a capacidade de leitura de palavras de baixa freqüência e de não-palavras. A leitura lexical está preservada e os pacientes podem ler palavras freqüentes (regulares e irregulares).

**Objetivo:**

Verificar a aplicação do modelo cognitivo na caracterização do distúrbio de leitura de uma paciente com dislexia adquirida e na elaboração e implementação de um programa de reabilitação.

**Métodos:**

Este estudo apresenta OCS, uma paciente de 57 anos, com dislexia do tipo fonológica adquirida após acidente vascular cerebral isquêmico temporo-parieto-occipital à esquerda. Um programa de reabilitação baseado nos princípios da Neuropsicologia Cognitiva foi elaborado. Não-palavras e palavras de baixa freqüência com tamanhos controlados foram usadas e a paciente era estimulada a ler os estímulos em voz alta em um tratamento realizado em 22 sessões.

**Resultados:**

A avaliação pós-teste mostrou melhoras quantitativas e qualitativas.Melhoras significativas foram verificadas no total de respostas corretas, incluindo autocorreções (p<0.01) e na leitura de palavras trissílabas e polissílabas de estrutura silábica simples (p=00007 e p=002 respectivamente).

**Conclusões:**

O uso do modelo cognitivo para propor um programa de reabilitação foi bem sucedido e pudemos observar melhora significativa das habilidades de leitura em um período curto de tratamento. As melhoras atingidas nesse período resultaram em desempenho funcional de leitura.

Although dyslexia is a common consequence of brain damage there are few studies about the rehabilitation of this disorder. Oral communication deficits have received more attention due to their major impact on the performance of daily activities In recent years, owing to advances in information technology, many activities that used to be carried out through face-to-face communication are being performed by written communication (eg e-mail communication, internet bank operations) and acquired dyslexia has become a much more disabling disorder. Therefore, it is necessary to study and report successful techniques and procedures aimed at reestablishing reading skills.

Cognitive Neuropsychology models of reading have been used to describe several syndromes of acquired dyslexia. Surface dyslexia is characterized by a preserved ability to read non-words and regularly spelled words in conjunction with a difficulty reading irregular words. Deep dyslexia is characterized by lexical reading with the production of semantic errors and a severe impairment of procedures of grapheme-phoneme conversion. In this disorder, only high frequency (mainly concrete) words are read efficiently. A third syndrome is phonological dyslexia in which the ability to derive sound from print non-lexically, is impaired and reading relies on lexical mechanisms^1-3^.

According to the Cognitive Neuropsychological Approach, an extensive evaluation to understand the preserved and impaired cognitive mechanisms is fundamental in order to devise an efficient rehabilitation plan. Subsequently, the program has to be implemented and its results then have to be measured^4,5^. Some studies have shown good results from therapies using this approach^6-10^.

The objective of this study was to verify the application of the cognitive model in the characterization of the reading disorder of a patient with acquired dyslexia and in the devising and implementation of a rehabilitation plan verifying its efficacy.

## Methods

### Subject

The study was conducted on a patient with acquired dyslexia (OCS) who belonged to the group of aphasic patients participating in a language rehabilitation program at the Neurolinguistics Program of Spech Therapy Course and Department of Neurology of Hospital das Clínicas, School of Medicine, University of São Paulo.

OCS was a 57-year-old, right-handed, Portuguese speaking woman with 4 years of formal education. Prior to brain damage, she had worked in sales and used to read frequently for her job and also as a hobby. She used writing for basic daily needs. In March of 1992, OCS suffered an ischemic stroke that resulted in complete right hemiparesis, right homonymous hemianopsia and aphasia. Computerized tomography scan showed extensive ischemic damage to the temporo-parieto-occipital structures of the left hemisphere.

One month after stroke, the patient was evaluated at the Speech Therapy Unit and global aphasia was diagnosed. The first two years post stroke she received treatment focused mainly on oral communication. Her oral comprehension and oral expression improved although she still presented some phonemic paraphasias, characterizing a conduction aphasia syndrome At this time, she discontinued the treatment Three years later, OCS decided to restart the speech therapy sessions regularly. Her main complaints concerned reading difficulties. She was evaluated using the Boston Diagnostic Aphasia Assessment (BDAE)^11^ and mild oral language impairments (anomia), dyslexia and agraphia were diagnosed. The right homonymous hemianopsia was completely compensated by this time and the patient could perform all of her daily activities independently with no external aids because her hemiparesia was very mild at this point.

### Material and procedures

The patient was submitted to a comprehensive reading evaluation. The protocol used was developed for the ***Human Frontier Science Program ***(HFSP)^12^ and consisted of an oral reading test composed of 190 stimuli distributed into regular and irregular words, non-words, short and long words, high and low frequency words, function words, abstract and concrete words and verbs. The protocol also contained complementary tests (lexical decision, matching a given oral definition to its written word to investigate the semantic system, reading letters aloud). Three complementary reading tests were developed to more precisely diagnose the type of dyslexia of OCS.

Complementary Test 1 (CT1) was used to verify the advantage in reading words over non-words (lexicality effect). It consisted of 40 non-words built with the same syllabic structures and length of 40 words from the HFSP protocol. This task was devised to complement the evaluation based on the HFSP protocol since its reading aloud list contains only ten non-words.

Complementary Test 2 (CT2) was used to evaluate the severity of the phonological disorder. It was composed of 80 non-words (20 monosyllabic, 20 disyllabic, 20 trisyllabic and 20 polysyllabic). Each of these groups consisted of ten non-words composed of a simple syllabic structure (consonant –vowel) and ten of a complex syllabic structure.

Complementary Test 3 (CT3) was used to characterize which syllabic groups OCS was unable to read. It was composed of a list of 220 monosyllables of varied syllabic structures in Portuguese.

According to the results of the cognitive reading evaluation, a treatment plan was devised, taking into account the preserved and impaired cognitive mechanisms.

The patient received individual speech therapy and the program was applied in 22 sessions of 50 minutes each (twice a week), over approximately four months. The sessions took place in a silent office at the Neurology Clinic of Hospital das Clínicas. Type of intervention and materials used will be described in Results – Devising of the Rehabilitation Program. The patient was evaluated every two months, in order to verify the effectiveness of the treatment Statistical analysis was conducted, through Chi-square tests. The value of significance accepted was 0.05.

## Results

### Reading evaluation - characterization of dyslexia in a cognitive approach

In the pre-test reading evaluation, OCS presented 86 correct responses (45.3%) on the list of words and nonwords to be read aloud from the HFSP protocol.

The errors included 38 responses where syllable–bysyllable reading was carried out (20%) and three self-correction attempts without success. Most of the patient's errors (70 errors) were literal paralexias (omissions, substitutions and transpositions of graphemes). In some cases the high number of substitutions for a target word led to changes such that this word could no longer be recognized. We classified these responses as non-word type errors (two errors). She also presented verbal paralexias ie the substitution of one word for another (five errors). In majority of instances, these substitutions were for words with structure (size, consonants and vowels) similar to the target word. These errors were classified as formal paralexias (22 errors) and can suggest an unsuccessful attempt to read an item lexically. Some less frequent errors were regularizations, ie reading an irregular word according to grapheme-to-phoneme conversion rules (one error) and stress errors, which consisted of improper pronunciation of a particular syllable in a word (one error).

**Figure 1:**
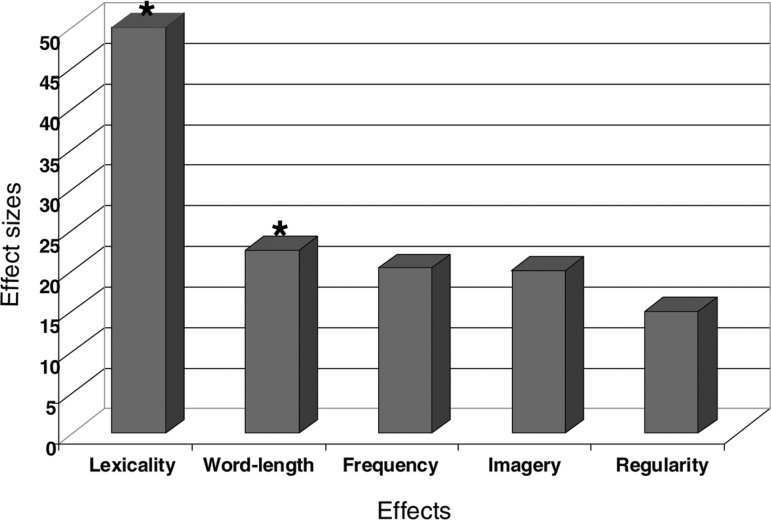
Analysis of linguistic variables on the pretest reading evaluation. Lexicality effect =% of correct responses to words minus % of correct responses to non-words; Word-length effect =% of correct responses to short stimuli minus % of correct responses to long stimuli; Frequency effect =% of correct responses to low frequency words minus % of correct responses to non-frequency words; Imagery effect =% of correct responses to abstract words minus % of correct responses to concrete words; Regularity effect =% of correct responses to irregular words minus % of correct responses to regular words * Significant effects: Lexicality (p <0.01) and word-length (p<0.05).

The patient presented the lexicality effect (significantly better performance on reading words compared to non-words, p<0.01) and the word-length effect (significantly better performance on reading short compared to long words, p<0.05). Other effects were not significant (Figure 1). Although not significant, there was a larger number of correct responses on regular than irregular words, on abstract than concrete words and on frequent than non-frequent words.

The results above (prevalence of literal paralexias, lexicality and word-length effects) showed that the difficulties of OCS were due to dysfunctional phonological reading. The patient's errors on irregular words were literal paralexias and not regularizations and occurred in low frequency irregular words. Irregular frequent words were read correctly. The larger number of correct responses in frequent words suggested the use of a relatively unimpaired lexical processing This syndrome is called phonological dyslexia.

Besides characterizing the dyslexia syndrome, the reading evaluation in a cognitive approach has the objective of verifying preserved and impaired cognitive mecha-nisms. Upon doing this, the therapist is able to better characterize the patient's dysfunction in order to devise a therapeutic program, drawing on the use of residual competences.

The patient’s performance on complementary tasks investigating lexical reading was relatively unimpaired OCS was able to distinguish words from non-words (90% of correct responses on the lexical decision test) and had preserved written comprehension for frequent words, suggesting preservation of the orthographic input lexicon and of the semantic system. However, she presented anomia, indicating a dysfunction of the phonological output lexicon.

On the complementary tasks investigating the phonological route, we observed that the patient could not segment syllables and graphemes properly, and was also unable to group letters into graphemes and graphemes into syllables.Moreover, grapheme-to-phoneme conversion mechanisms were impaired.

### Devising of the rehabilitation program

***Step 1: Determining the severity of dysfunction of phonological reading -complementary tests*** – Through the reading evaluation with the HFSP protocol it was possible to diagnose the dyslexia type presented by the patient and to determine the impaired and preserved cognitive mechanisms.

As the patient presented phonological dyslexia, one possible therapeutic strategy could be the rehabilitation of the phonological route. Another could be the training of a group of words to build more representations to favor lexical reading.We chose the former option because the patient presented some regularization and stress errors (although very few), demonstrating that phonological reading was not completely abolished.Moreover, the Portuguese language has very few irregularities for reading and with developed phonological skills almost all words can be read properly. For these reasons we believed that the first strategy would yield more benefit for the patient.

The first step was to determine which stimuli could be properly read with the residual competence of the phonological route. By determining the extension and syllabic complexity of the correctly read stimuli we would be able to plan the several stages of the therapy.

Therefore, CT2 and CT3 were devised to assess these elements. They were used to characterize the level of impairment of the phonological route of reading.

Through CT2 we were able to verify that the only stimuli read correctly, non-lexically, by the patient were monosyllables of simple syllabic structure (consonantvowel- CV) (80% of correct responses, or 100% considering self-correction attempts).When word extension and syllabic complexity increased, the number of correct responses was reduced or nonexistent. Therefore, with two-syllable non-words of simple syllabic structure her performance decreased to 40% whilst three-syllable stimuli could not be read at all.With complex syllabic structure only 10% of the monosyllabic and disyllabic stimuli were correctly read.

A higher difficulty with complex syllabic structures was confirmed with CT3. The patient achieved 93.3% of correct responses in simple syllabic structures (CV) and 43% on complex ones such as VC, VV, CVV, CVC and CCV.

***Step 2: Planning and implementing the program*** – From the results obtained from this comprehensive evaluation, a therapeutic plan was devised with the purpose of recovering non-lexical reading.We used a set of nonwords and low frequency words for the treatment. These stimuli necessarily require non-lexical reading. The stimuli used for therapy did not include words and nonwords belonging to the pre-test/post-test evaluation protocol. Our main strategies were the following:

1. To promote awareness of the CV syllabic structure, through activities involving syllabic division. This would help the patient to recover the ability to segment and group syllables.
2. To motivate syllable-by-syllable reading. In the beginning of the treatment only, two-syllable non-words and low-frequency words, composed of simple syllabic structures were used.

According to the patient’s progress, longer stimuli were used and some complex syllabic structures were introduced. In the tenth session OCS began to present systematic successes in trisyllabic stimuli of simple syllabic structure. Next, besides including practice using polysyllabic stimuli with simple syllabic structure, we started promoting awareness of complex syllabic structures (VC, CVC and CCV).

We asked the patient to do some practice at home but up to the 12^th^ session this training was restricted to low frequency words.

To intensify the use of the phonological route, on the 13^th^ session we started using some lists of monosyllabic stimuli with several syllabic structures and asked the patient to read them as fast as she could. At this point, the patient became really motivated with her progress and increased practice time at home, and we were then able to include non-word reading in her home practice As a result, her reading speed improved, especially for two-syllable stimuli that were no longer read syllable-by-syllable.

### Treatment results - evaluation of the efficacy of the therapeutic program

The first revaluation was done after ten sessions of treatment and the second, after 22.

**Figure 2:**
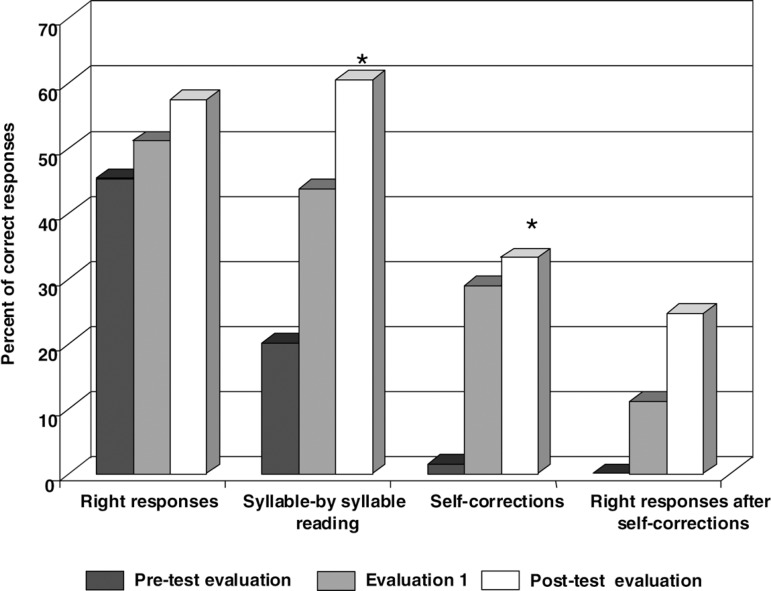
Percent of correct responses of OCS on HFSP oral reading test.

The post-test evaluation showed quantitative and qualitative improvements (Figure 2). On the HFSP protocol, we observed an increase in the number of correct responses, the number of words read syllable-by-syllable and the attempts at self-correction. The percentage of correct responses increased to 57.4%.When correct responses after self-correction attempts were considered, this percentage totaled 82.1%, representing a significant improvement (p<0.01). The higher accuracy on reading, even considering self-corrections, resulted in better reading comprehension The percentage of stimuli for which the patient accomplished syllable-by-syllable reading increased from 20% to 60.5% (p<0.01) and the percentage of self-corrections increased from 15% to 33.2% (p< 0.01). These data indicates an increased occurrence of non-lexical reading. The overall performance indicates an improvement on grapheme-to-phoneme conversion procedures.

**Figure 3:**
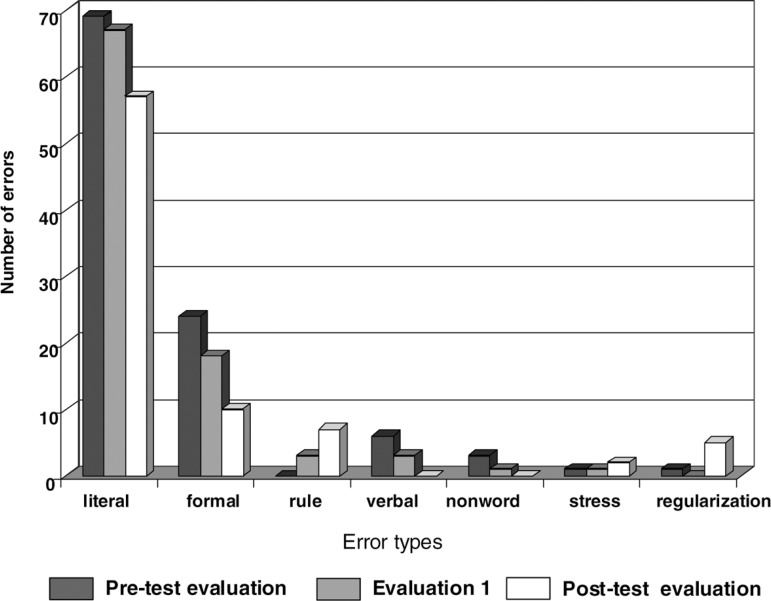
Types of errors of OCS on the HFSP oral reading test.

Statistical analysis did not reach significance regarding the amount and type of paralexias In spite of this, we were able verify that although literal errors were still very prevalent, their number decreased and the patient's responses presented more similarity with the target words, demonstrating an improvement in the conversion procedure. Despite being few in number, the increase in errors on the application of rules as well as stress errors are noteworthy because they denote the use of the phonological strategy for reading. Therefore, when syllableby- syllable reading started to be more used by the patient, such errors became more frequent (Figure 3).

Regarding linguistic variables, there was not much change in the effects observed on the evaluation: the patient continued to exhibit higher levels of difficulty in reading long low frequency words with complex syllabic structures.

The progress of non-lexical reading can be seen in Table 1. Improvements were verified in the reading of non-words of simple syllabic structure. The improvements in trisyllabic and polysyllabic stimuli were significant (p=0.07 and p=0.02 respectively). Improvements in reading non-words of complex syllabic structure were verified but the results were not significant. This can be explained by the fact that just some complex syllabic structures were introduced in the treatment (VC / CVC / CCV).

**Table 1:** Performance of OCS on the Complementary Test 2 - Efficacy of the treatment.

	Pre-test evaluation	Evaluation 1	Post-test evaluation
	(% of correct responses)	(% of correct responses)	(% of correct responses)
MS-SSS	80	100	100
Self-corrections on MS-SSS	100	100	100
DS-SSS	40	60	80
Self-corrections on DS-SSS	40	90	90
TS-SSS	0	20	60**
Self-corrections on TS-SSS	0	80	90
			
PS-SSS	0	0	40*
Self-corrections on PS-SSS	20	20	70
MS-CSS	10	10	30
Self-corrections on MS-CSS	20	20	50
DS-CSS	10	20	50
Self-corrections on DS-CSS	30	40	60
TS-CSS	0	0	20
Self-corrections on TS-CSS	0	10	50
PS-CSS	0	0	10
Self-corrections on TS-CSS	0	0	20

MS, monosyllabic stimuli; DS, disyllabic stimuli; TS, trisyllabic stimuli; PS, polysyllabic stimuli; SSS, simple syllabic structure; CSS, complex syllabic structure;

* Significant improvement (Chi-square test, p<0.005);

** Significant improvement (Chi-square test, p<0.01).

With the improvements achieved, the patient improved her performance on daily activities and was satisfied. She considered she was able to read “enough” for her needs. Therefore, she decided to discontinue the treatment the following year.

## Discussion

This study was aimed at checking the application of the cognitive model in the characterization of the reading disorder of a patient with acquired dyslexia and in the devising and implementation of a rehabilitation plan.

Regardless of the approach of a rehabilitation program, three different milestones characterize the language rehabilitation of brain-damaged patients: the evaluation, the therapy and the end of the treatment, when goals are achieved or therapy is no longer effective. The cognitive approach presents the best option regarding coherence among these milestones^13^. Therapists often perform an evaluation whose purpose is to classify the type of aphasia (focused on syndrome classification, from an anatomo- clinical perspective) and then plan a therapy based on the use of language (eg developing alternative communication). There is no problem with this kind of evaluation or with the therapy procedures themselves but there is a lack of coherence between these two points. On the other hand, the comprehensive evaluation proposed by Cognitive Neuropsychology can provide more elements to therapy planning. It is aimed at precisely identifying the impaired and preserved cognitive mechanisms involved in the execution of a given function, which can result in more specific and goal-driven treatments with faster results.

In the case of OCS, a syndrome evaluation showed that she presented dyslexia.Within a cognitive approach, we could define that lexical reading was relatively preserved and that the treatment needed to focus on grapheme- to-phoneme conversion procedures. The devising of complementary tests helped us to define the starting point. Therefore, we consider that although the evaluation was longer, it resulted in a shorter treatment.

In addition, this precise evaluation yielded evidence suggesting treatment efficacy.We consider that the results obtained were due to treatment for two reasons. First, the patient was outside the period of spontaneous recovery. Second, the improvements were obtained only when the developed strategy was used OCS did not improve reading on all kinds of linguistic variables. In fact, her performance decreased on irregular words, because she started to read non-lexically. This suggests that the treatment was really effective in its purposes and that the results were not due to other nonspecific factors.

Our patient considered that she was able to perform her daily activities following the progress she had made up to that point. However, we had a plan to continue the treatment if she had wished to. As the whole therapeutic work had motivated non-lexical reading, it was expected that when phonological reading was totally reestablished the patient would present regularization, rule and stress errors, characterizing the surface dyslexia syndrome. At this point, it would be important to focus on the work with contextual rules and irregular words. Later, work with sentences and texts should be carried out.

To sum up, we consider that the use of the cognitive model to determine the specific impairments on reading processing, as well as for devising a therapeutic plan and for treatment efficacy assessment was valid and appropriate for this patient. Through the cognitive approach it was possible to act specifically on the dysfunctional processing, achieving important improvements in a reduced number of sessions.

We hope this work might contribute toward the verification of the validity of applying the cognitive model in the therapeutic approach. Other cases have been reported with different therapeutic strategies to reading rehabilitation, but similar principles for evaluation and treatment proposal, resulting in benefits to the patients^6-10,14-15^. However, further studies need to be conducted to confirm our results. The challenge is to have more cases published in order to find regularities and to more accurately define successful treatment techniques.
